# Editorial: Stem cell-derived retinal and brain organoid culture for disease modeling

**DOI:** 10.3389/fncel.2024.1367482

**Published:** 2024-03-13

**Authors:** Lin Cheng, Maeve Ann Caldwell, Kin-Sang Cho, Carla B. Mellough

**Affiliations:** ^1^Department of Ophthalmology and Visual Sciences, University of Iowa Carver College of Medicine, Iowa City, IA, United States; ^2^Center for the Prevention and Treatment of Visual Loss, Veterans Affairs Medical Center, Iowa City, IA, United States; ^3^Discipline of Physiology, Trinity College Institute for Neuroscience, Trinity College Dublin, Dublin, Ireland; ^4^Schepens Eye Research Institute of Massachusetts Eye and Ear, Harvard Medical School, Boston, MA, United States; ^5^Centre for Ophthalmology and Visual Sciences (incorporating Lions Eye Institute), The University of Western Australia, Nedlands, WA, Australia

**Keywords:** retinal organoids (ROs), brain organoids (BOs), stem cells, central nervous system (CNS) diseases, neurodegenerative disorders

CNS organoids are an emerging research tool in stem cell biology to recapitulate retinal and brain development and can be used for studying disease mechanisms, regenerative medicine, precision medicine, and cell treatment. Retinal organoids (ROs) consist of neural retina and retinal pigment epithelium (RPE). Brain organoids (BOs) consist of multiple neural lineage cells and contain fluid-filled ventricle-like structures surrounded by a ventricular/subventricular (VZ/SVZ) zone-like layer of neural stem cells (NSCs). The BO culture protocol could be further refined to culture-specific brain regions such as the cerebral cortex, forebrain, midbrain, hindbrain, brainstem, choroid plexus, cerebellum, thalamus, spinal cord, etc., in organoids. This editorial article frames the aim of this Research Topic as gathering promising, recent, and novel research trends on modeling central nervous system (CNS) diseases using the retinal and brain organoids.

There are two main approaches to establishing ROs (Cheng and Kuehn, 2023) and many approaches for generating different regions of BOs (Mayhew and Singhania, 2023). The modification and improvement of these methods are continuously ongoing. One advantage of ROs and BOs is that we can control the specificity of cell populations and maturity by timing the culture system.

For the 3-dimensional (3D) organoids, quantifying and analyzing multidimensional data for the complex *in vitro* models (organoids, organ-on-chip) is difficult. Kegeles et al.'s paper introduced a deep learning-based computer algorithm to recognize and predict RO differentiation based on bright-field imaging. This paper leverages a machine-learning tool (convolutional neural networks, CNNs) to analyze images. Image acquisition and analysis are universal, robust, and non-invasive and can generate quantitative, decision-making data to assess retinal differentiation without chemical probes or reporter gene expression.

The patient-derived organoids can be grown to model developmental diseases. Eintracht et al. and Vielle et al. reviewed the use of human induced pluripotent stem cells (hiPSC)-derived ROs to model developmental eye disorders such as microphthalmia caused by a VSX2 variant. Eintracht et al. also reviewed the spatiotemporal gene expression patterns and interactions between the embryonic germ layers during vertebrate eye development. Vielle et al. reviewed the limitations of the ROs to model eye developmental disorders, such as RPE not juxtaposed to the apical side of the neural retina, no optic nerve (retinal ganglion cells dying as ROs mature), no macula formation, lack of microglia, no yolk sac derivatives that invade the optic cup during the period of retinogenesis *in vivo*, and absence blood vessels. ROs are neuroectodermal derivatives and will not have mesodermal original cells or tissues.

ROs can be an effective platform for identifying molecular therapeutic targets and for future clinical applications. Singh and Nasonkin pointed out that the implicit limitation of ROs is the absence of a uniform layer of RPE and the direct exposure of most photoreceptors in the developing organoids to neural medium. Li et al. reviewed the methods to augment RO production, reduction of RO heterogeneity, and transplantation of RO-derived retinal ganglion cells and photoreceptors. Garita-Hernandez et al. generated a hiPSC line with enhanced gene expression in cone cells using a 1.7-kb L-opsin promoter. They fused the red-shifted opsin Jaws with a fluorescent reporter gene driven by an L-opsin promoter, enabling enriched cell sorting of the cone cells. This study underscores the importance of promoter activity in restricting, improving, and controlling the kinetics of transgene expression during the maturation of ROs. Ning et al. explored an approach to selecting and expanding the Müller glial cells (MGCs) from hiPSC-derived ROs. ROs older than 120 days are an optimal source for enriching MGCs with high purity and expansion ability. MGCs could be passaged at least 10 times serially, yielding large numbers of cells in a relatively short period of time.

Speaking of BOs, cortical organoids can be used to model Alzheimer's disease (AD) in Down syndrome (DS). Zhao and Haddad found DS patient iPSC-derived cortical organoids have much higher amyloid beta (Aß) immunoreactivity, a significantly higher number of amyloid plaques, an increased Tau phosphorylation (pTau S396) and insoluble Tau/total Tau in DS organoids than control organoids. They found the signature of AD is present in DS much earlier than predicted, even in early fetal brain development.

Parkinson's disease (PD) is a progressive neurodegenerative disease, being the second most common after AD (Parkinson's Foundation, 2023). Midbrain dopaminergic (DA) neurons are selectively lost in PD. The presynaptic neuronal protein alpha-synuclein (α-syn), which tends to accumulate and aggregate in PD brains as Lewy bodies, is the key driver of PD. Therefore, preventing the propagation of α-syn could slow PD progression. However, multiple cell types, such as astrocytes, microglia, and oligodendrocytes, are involved in α-syn deposition. Koh et al. proposed midbrain organoids are a promising platform for investigating α-syn accumulation, aggregation, and transmission in PD due to (1) dopaminergic neurons in organoids are far more mature than those in 2D; (2) genetically resembling the prenatal midbrain; (3) midbrain organoids contained spatially organized groups of dopaminergic neurons with other neuronal, astroglial, and oligodendrocyte cells also being differentiated, which could permit the study of how these cell types interplay with each other; and (4) they contain both mature and aged neurons, which is more clinically relevant. The authors also proposed to co-culture PD iPSC-derived neurons with healthy neurons, either in 2D or as organoids, to study α-syn propagation.

Since Lancaster et al. (2013) reported the first method of human cerebral organoids containing ventricles and neural layers of the cerebral cortex, a great effort has been put into human BO development and disease modeling. However, neural circuit formation with BOs has rarely been investigated. A paper published in this Research Topic raises the challenges in modeling human neural circuit formation using the BOs. Matsui et al. used fused organoids to study the neural circuit, such as glutamatergic excitatory neuron-rich cerebral organoids fused with GABAergic interneuron-rich subpallium organoids to model neuronal migration between distant components. Human cerebral organoids fused with thalamic organoids are also used to model the thalamocortical circuit involved in transmitting sensory and motor information in the human brain. They pointed out that the current organoids possess multiple randomly positioned neural tube-like structures and, therefore, lack a fixed structural axis. As such, organoids don't have gradients of molecules that are required for proper neuronal migration, axonal guidance, and synapse formation that leads to circuit formation. The authors also stressed that the myeloid cells (such as microglia, vascular endothelial cells, and pericytes) in BOs might help study vasculature-mediated neural circuit formation.

To overcome organoid-to-organoid variability and batch-to-batch variation, Eigenhuis et al. developed a simplified protocol for the robust and reproducible generation of cortical organoids from hiPSC. The cortical organoids contain apical radial glial and intermediate progenitors, deep and upper layer neurons, and astrocytes. Unlike the “self-patterned” generation of cortical organoids, the authors used exogenous patterning factors to guide or direct region-specific identities in organoids.

In summary, ROs and BOs are promising and powerful research tools in CNS disease modeling. However, there are still challenges and limitations of ROs and BOs in the application that need to be tackled. Integrating with other technologies, such as organ-on-chip, omics, live imaging, machine learning, artificial intelligence, and asssembloids (different organoids co-cultured together), will broaden the utilization of ROs and BOs and provide unprecedented insight into CNS diseases.

## Author contributions

LC: Writing—original draft. MC: Writing—review & editing. K-SC: Writing—review & editing. CM: Writing—review & editing.
